# Supplementary material for: Unveiling the Bioactive Potential of the Invasive Jellyfish Phyllorhiza punctata Through Integrative Transcriptomic and Proteomic Analyses
Source: Biomolecules. 2025 Aug 4;15(8):1121. doi: 10.3390/biom15081121 (PMC12383608; doi:10.3390/biom15081121)
Supplement: Supplementary file 1 [file biomolecules-15-01121-s001.zip › Suplementary Material/FileS3/PEPFOLD4/XP_065069917_715/clusters.html]

 PEP-FOLD clusters


Download archive of all models  here   

|  |  |  |
| --- | --- | --- |
| # | file | sOPEP |
| # cluster 1 - Size: 1 |  |  |
|  | XP\_065069917\_715-model1 | -36.7139 |
| # cluster 2 - Size: 3 |  |  |
|  | XP\_065069917\_715-model2 | -34.2021 |
|  | XP\_065069917\_715-model2.1 | -29.186 |
|  | XP\_065069917\_715-model2.2 | -27.1772 |
| # cluster 3 - Size: 1 |  |  |
|  | XP\_065069917\_715-model3 | -34.0074 |
| # cluster 4 - Size: 1 |  |  |
|  | XP\_065069917\_715-model4 | -33.8138 |
| # cluster 5 - Size: 2 |  |  |
|  | XP\_065069917\_715-model5 | -32.8681 |
|  | XP\_065069917\_715-model5.1 | -31.3591 |
| # cluster 6 - Size: 1 |  |  |
|  | XP\_065069917\_715-model6 | -32.6066 |
| # cluster 7 - Size: 1 |  |  |
|  | XP\_065069917\_715-model7 | -30.4985 |
| # cluster 8 - Size: 1 |  |  |
|  | XP\_065069917\_715-model8 | -30.1997 |
| # cluster 9 - Size: 1 |  |  |
|  | XP\_065069917\_715-model9 | -29.5287 |
| # cluster 10 - Size: 1 |  |  |
|  | XP\_065069917\_715-model10 | -29.5087 |
| # cluster 11 - Size: 2 |  |  |
|  | XP\_065069917\_715-model11 | -29.0518 |
|  | XP\_065069917\_715-model11.1 | -27.0203 |
| # cluster 12 - Size: 1 |  |  |
|  | XP\_065069917\_715-model12 | -28.4933 |
| # cluster 13 - Size: 5 |  |  |
|  | XP\_065069917\_715-model13 | -28.3817 |
|  | XP\_065069917\_715-model13.1 | -22.34 |
|  | XP\_065069917\_715-model13.2 | -22.1804 |
|  | XP\_065069917\_715-model13.3 | -22.0827 |
|  | XP\_065069917\_715-model13.4 | -21.2934 |
| # cluster 14 - Size: 1 |  |  |
|  | XP\_065069917\_715-model14 | -28.2812 |
| # cluster 15 - Size: 1 |  |  |
|  | XP\_065069917\_715-model15 | -28.1277 |
| # cluster 16 - Size: 1 |  |  |
|  | XP\_065069917\_715-model16 | -27.9327 |
| # cluster 17 - Size: 2 |  |  |
|  | XP\_065069917\_715-model17 | -27.5704 |
|  | XP\_065069917\_715-model17.1 | -27.2817 |
| # cluster 18 - Size: 1 |  |  |
|  | XP\_065069917\_715-model18 | -27.5595 |
| # cluster 19 - Size: 1 |  |  |
|  | XP\_065069917\_715-model19 | -27.4296 |
| # cluster 20 - Size: 1 |  |  |
|  | XP\_065069917\_715-model20 | -27.3821 |
| # cluster 21 - Size: 2 |  |  |
|  | XP\_065069917\_715-model21 | -27.3213 |
|  | XP\_065069917\_715-model21.1 | -24.7525 |
| # cluster 22 - Size: 2 |  |  |
|  | XP\_065069917\_715-model22 | -27.2552 |
|  | XP\_065069917\_715-model22.1 | -26.9052 |
| # cluster 23 - Size: 5 |  |  |
|  | XP\_065069917\_715-model23 | -27.2041 |
|  | XP\_065069917\_715-model23.1 | -26.7639 |
|  | XP\_065069917\_715-model23.2 | -24.3978 |
|  | XP\_065069917\_715-model23.3 | -23.078 |
|  | XP\_065069917\_715-model23.4 | -23.0534 |
| # cluster 24 - Size: 1 |  |  |
|  | XP\_065069917\_715-model24 | -27.027 |
| # cluster 25 - Size: 2 |  |  |
|  | XP\_065069917\_715-model25 | -26.692 |
|  | XP\_065069917\_715-model25.1 | -22.7917 |
| # cluster 26 - Size: 1 |  |  |
|  | XP\_065069917\_715-model26 | -26.1738 |
| # cluster 27 - Size: 1 |  |  |
|  | XP\_065069917\_715-model27 | -26.1695 |
| # cluster 28 - Size: 1 |  |  |
|  | XP\_065069917\_715-model28 | -26.0627 |
| # cluster 29 - Size: 1 |  |  |
|  | XP\_065069917\_715-model29 | -25.4754 |
| # cluster 30 - Size: 1 |  |  |
|  | XP\_065069917\_715-model30 | -25.4559 |
| # cluster 31 - Size: 1 |  |  |
|  | XP\_065069917\_715-model31 | -25.3492 |
| # cluster 32 - Size: 1 |  |  |
|  | XP\_065069917\_715-model32 | -25.2774 |
| # cluster 33 - Size: 2 |  |  |
|  | XP\_065069917\_715-model33 | -25.208 |
|  | XP\_065069917\_715-model33.1 | -22.8546 |
| # cluster 34 - Size: 1 |  |  |
|  | XP\_065069917\_715-model34 | -24.631 |
| # cluster 35 - Size: 2 |  |  |
|  | XP\_065069917\_715-model35 | -24.5548 |
|  | XP\_065069917\_715-model35.1 | -22.8386 |
| # cluster 36 - Size: 2 |  |  |
|  | XP\_065069917\_715-model36 | -24.4884 |
|  | XP\_065069917\_715-model36.1 | -23.6292 |
| # cluster 37 - Size: 1 |  |  |
|  | XP\_065069917\_715-model37 | -24.4105 |
| # cluster 38 - Size: 2 |  |  |
|  | XP\_065069917\_715-model38 | -24.3347 |
|  | XP\_065069917\_715-model38.1 | -21.5077 |
| # cluster 39 - Size: 1 |  |  |
|  | XP\_065069917\_715-model39 | -24.2682 |
| # cluster 40 - Size: 1 |  |  |
|  | XP\_065069917\_715-model40 | -24.2528 |
| # cluster 41 - Size: 3 |  |  |
|  | XP\_065069917\_715-model41 | -23.7164 |
|  | XP\_065069917\_715-model41.1 | -23.6314 |
|  | XP\_065069917\_715-model41.2 | -22.567 |
| # cluster 42 - Size: 1 |  |  |
|  | XP\_065069917\_715-model42 | -23.6352 |
| # cluster 43 - Size: 1 |  |  |
|  | XP\_065069917\_715-model43 | -23.2273 |
| # cluster 44 - Size: 1 |  |  |
|  | XP\_065069917\_715-model44 | -23.1139 |
| # cluster 45 - Size: 10 |  |  |
|  | XP\_065069917\_715-model45 | -22.9097 |
|  | XP\_065069917\_715-model45.1 | -22.8757 |
|  | XP\_065069917\_715-model45.2 | -22.7858 |
|  | XP\_065069917\_715-model45.3 | -22.1751 |
|  | XP\_065069917\_715-model45.4 | -21.7551 |
|  | XP\_065069917\_715-model45.5 | -21.7067 |
|  | XP\_065069917\_715-model45.6 | -21.4803 |
|  | XP\_065069917\_715-model45.7 | -21.222 |
|  | XP\_065069917\_715-model45.8 | -21.175 |
|  | XP\_065069917\_715-model45.9 | -20.1337 |
| # cluster 46 - Size: 1 |  |  |
|  | XP\_065069917\_715-model46 | -22.8308 |
| # cluster 47 - Size: 1 |  |  |
|  | XP\_065069917\_715-model47 | -22.8032 |
| # cluster 48 - Size: 1 |  |  |
|  | XP\_065069917\_715-model48 | -22.7938 |
| # cluster 49 - Size: 2 |  |  |
|  | XP\_065069917\_715-model49 | -22.5891 |
|  | XP\_065069917\_715-model49.1 | -22.558 |
| # cluster 50 - Size: 1 |  |  |
|  | XP\_065069917\_715-model50 | -22.5802 |
| # cluster 51 - Size: 1 |  |  |
|  | XP\_065069917\_715-model51 | -22.1919 |
| # cluster 52 - Size: 1 |  |  |
|  | XP\_065069917\_715-model52 | -22.0196 |
| # cluster 53 - Size: 1 |  |  |
|  | XP\_065069917\_715-model53 | -21.784 |
| # cluster 54 - Size: 1 |  |  |
|  | XP\_065069917\_715-model54 | -21.6733 |
| # cluster 55 - Size: 1 |  |  |
|  | XP\_065069917\_715-model55 | -21.6651 |
| # cluster 56 - Size: 1 |  |  |
|  | XP\_065069917\_715-model56 | -21.593 |
| # cluster 57 - Size: 1 |  |  |
|  | XP\_065069917\_715-model57 | -21.42 |
| # cluster 58 - Size: 1 |  |  |
|  | XP\_065069917\_715-model58 | -21.4136 |
| # cluster 59 - Size: 6 |  |  |
|  | XP\_065069917\_715-model59 | -21.2158 |
|  | XP\_065069917\_715-model59.1 | -21.1865 |
|  | XP\_065069917\_715-model59.2 | -21.0888 |
|  | XP\_065069917\_715-model59.3 | -20.5438 |
|  | XP\_065069917\_715-model59.4 | -20.3364 |
|  | XP\_065069917\_715-model59.5 | -19.3845 |
| # cluster 60 - Size: 1 |  |  |
|  | XP\_065069917\_715-model60 | -20.8461 |
| # cluster 61 - Size: 1 |  |  |
|  | XP\_065069917\_715-model61 | -20.7855 |
| # cluster 62 - Size: 1 |  |  |
|  | XP\_065069917\_715-model62 | -20.2001 |
| # cluster 63 - Size: 1 |  |  |
|  | XP\_065069917\_715-model63 | -19.8796 |
|  |  |  |
